# Supplementary material for: Product Validation and Stability Testing of Pharmacy Compounded Cholic Acid Capsules for Dutch Patients with Rare Bile Acid Synthesis Defects
Source: Pharmaceutics. 2023 Feb 26;15(3):773. doi: 10.3390/pharmaceutics15030773 (PMC10059964; doi:10.3390/pharmaceutics15030773)
Supplement: Supplementary file 1 [file pharmaceutics-15-00773-s001.zip › pharmaceutics-2163972-supplementary.pdf]

Product validation and stability testing of pharmacy compounded cholic acid capsules for Dutch patients with rare bile acid synthesis defects

Supplementary Material

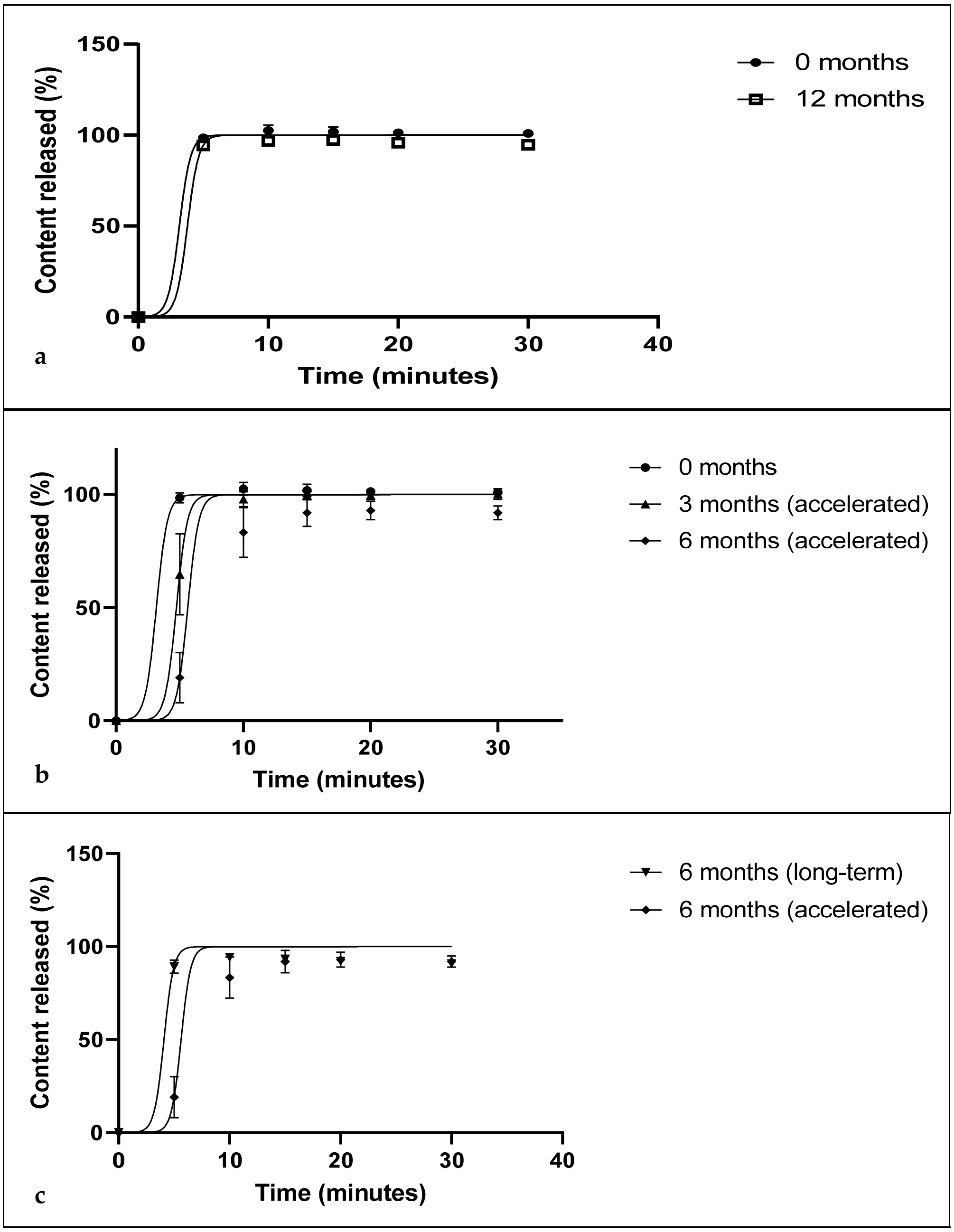

Figure S1. Dissolution profiles of 25 mg CA capsules: (**a**) Dissolution profile of 25 mg CA capsule at T0 vs. T12, stored under long-term storage conditions (25°C ± 2°C / 60% ± 5% RH); (**b**) Dissolution profile of 25 mg CA capsule at T0 vs. T3 and T6, stored under accelerated storage conditions (40°C ± 2°C / 75% ± 5% RH) ; (**c**) Dissolution profile of 25 mg CA capsule at T6 stored under long-term (25°C ± 2°C / 60% ± 5% RH) vs. accelerated storage conditions (40°C ± 2°C / 75% ± 5% RH).

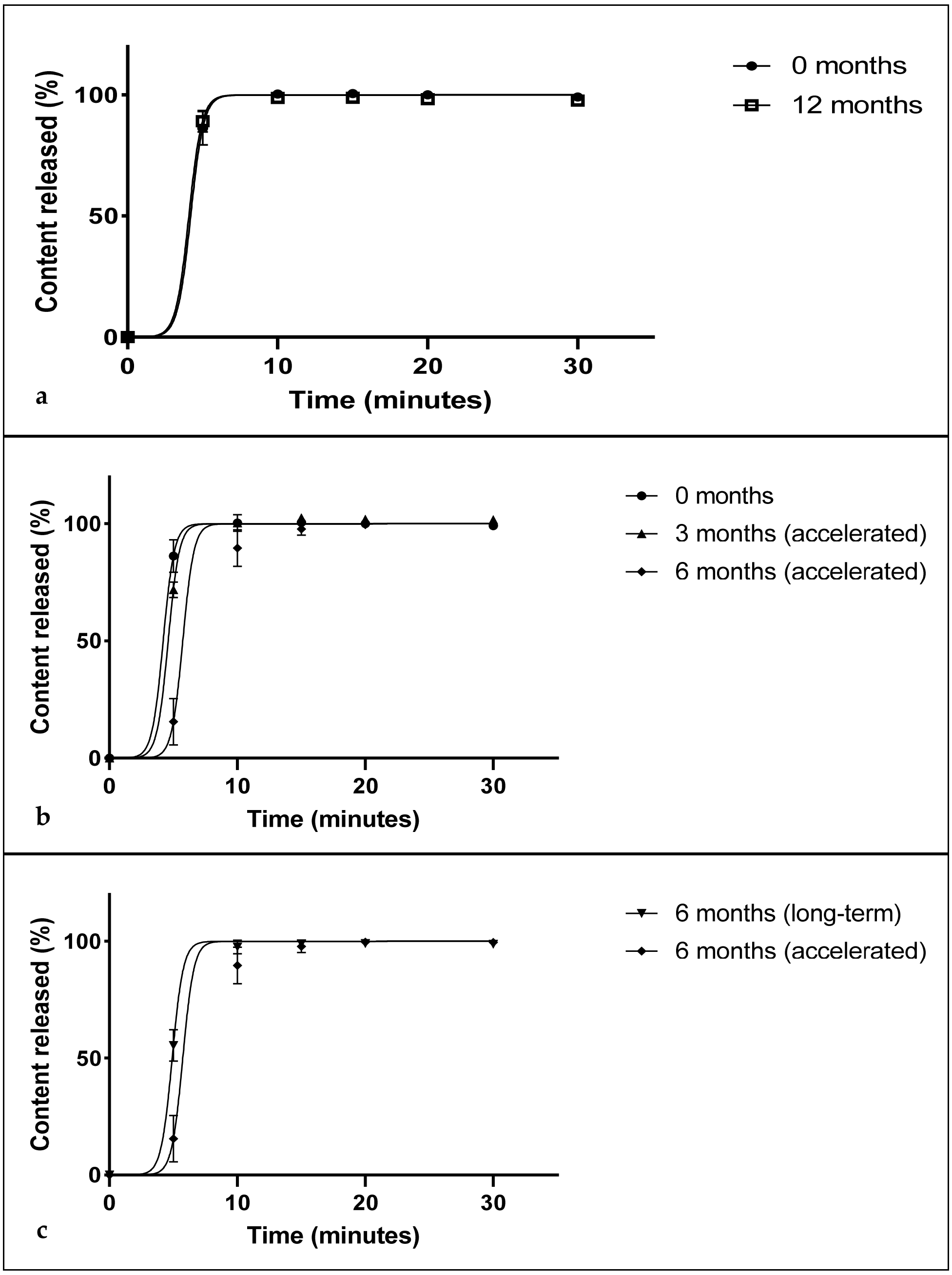

Figure S2. Dissolution profiles of 250 mg CA capsules: (**a**) Dissolution profile of 250 mg CA capsule at T0 vs. T12, stored under long-term storage conditions (25°C ± 2°C / 60% ± 5% RH) ; (**b**) Dissolution profile of 250 mg CA capsule at T0 vs. T3 and T6, stored under accelerated storage conditions (40°C ± 2°C / 75% ± 5% RH) ; (**c**) Dissolution profile of 250 mg CA capsule at T6 stored under long-term (25°C ± 2°C / 60% ± 5% RH) vs. accelerated storage conditions (40°C ± 2°C / 75% ± 5% RH).

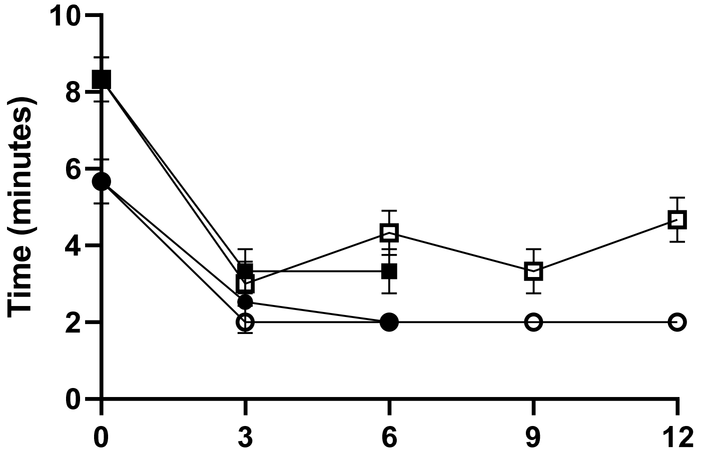

Figure S3. Disintegration times of 25 and 250 mg CA capsules stored over time under long-term conditions (25°C ± 2°C / 60% ± 5% RH) and accelerated (stressed) storage conditions (40°C ± 2°C / 75% ± 5% RH) (mean ± SD, n=3). o 25 mg CA capsule under long-term storage condition; ● 25 mg CA capsule under accelerated storage condition; □ 250 mg CA capsule under long-term storage condition; ■ 250 mg CA capsule under accelerated storage condition.

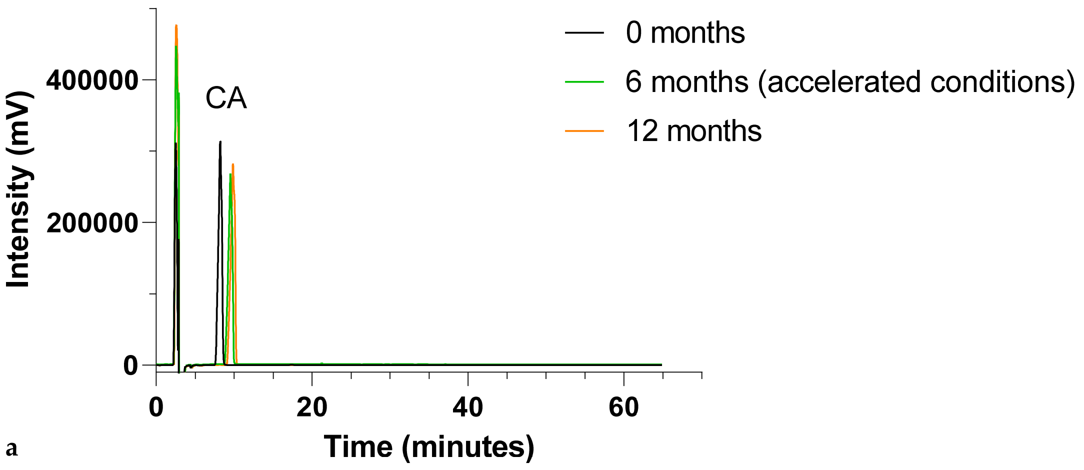

a

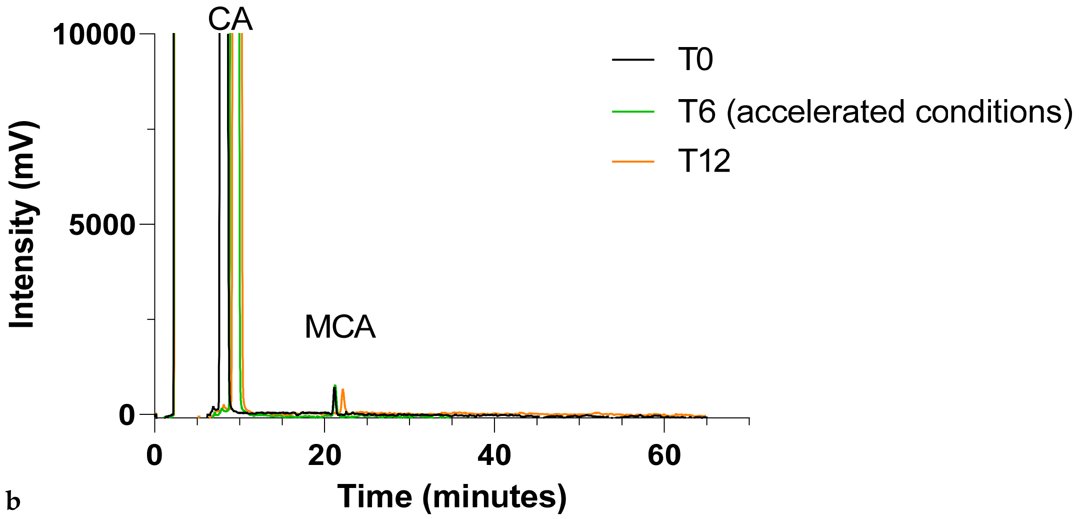

b

Figure S4. Representative chromatograms of related substance HPLC-RI analysis of 25 mg CA capsules at T0 and T12, stored under long-term storage conditions (25°C ± 2°C / 60% ± 5% RH), and T6, stored under accelerated (stressed) storage conditions (40°C ± 2°C / 75% ± 5% RH). HPLC-RI analysis using RID-10A detector and a reversed phase X-bridge BEH phenyl column 250 x 4.6 x 3.5 µm (30˚C ± 2˚C), injection volume: 30 µL, flow rate: 1.0 mL/min, mobile phase: 30 mmol/L potassium dihydrogen phosphate (KH2PO4) and 600 µL phosphoric acid in a 1 liter mixture of water/acetonitril (ACN)(60:40); (**a**) CA peaks at ±8,3 minutes; (**b**) MCA peaks at ± 22 minutes (chromatogram enlarged).

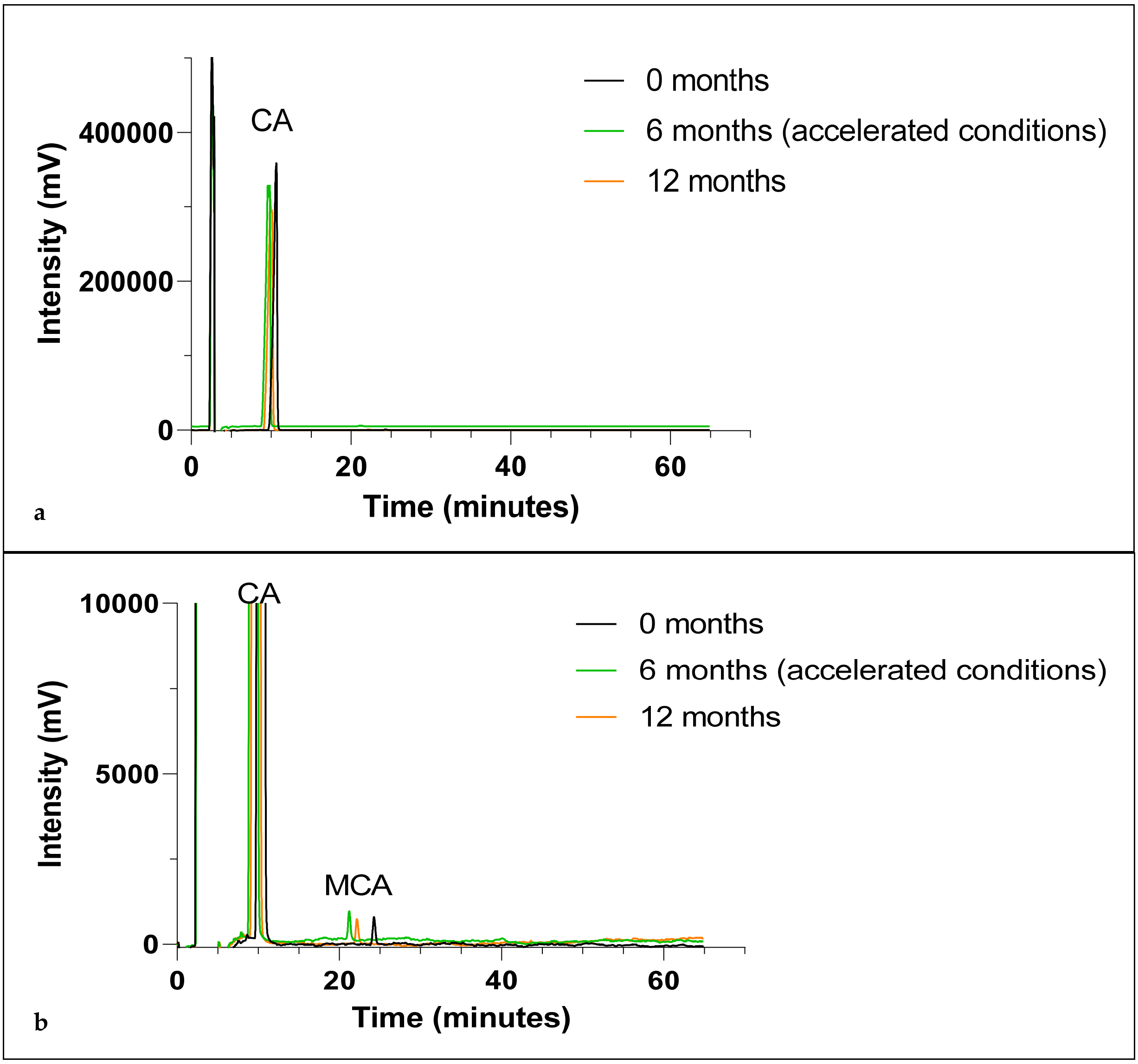

Figure S5. Representative chromatograms of related substance HPLC-RI analysis of 250 mg CA capsules at T0 and T12, stored under long-term storage conditions (25°C ± 2°C / 60% ± 5% RH), and T6, stored under accelerated (stressed) storage conditions (40°C ± 2°C / 75% ± 5% RH). HPLC-RI analysis using RID-10A detector and a reversed phase X-bridge BEH phenyl column 250 x 4.6 x 3.5 µm (30˚C ± 2˚C), injection volume: 30 µL, flow rate: 1.0 mL/min, mobile phase: 30 mmol/L potassium dihydrogen phosphate (KH2PO4) and 600 µL phosphoric acid in a 1 liter mixture of water/acetonitril (ACN)(60:40); (**a**) CA peaks at ±8,3 minutes; (**b**) MCA peaks at ± 22 minutes (chromatogram enlarged).
